# Wetting Induced Oxidation of Pt-based Nano Catalysts Revealed by *In Situ* High Energy Resolution X-ray Absorption Spectroscopy

**DOI:** 10.1038/s41598-017-00639-1

**Published:** 2017-05-03

**Authors:** Yi-Tao Cui, Yoshihisa Harada, Hideharu Niwa, Tatsuya Hatanaka, Naoki Nakamura, Masaki Ando, Toshihiko Yoshida, Kenji Ishii, Daiju Matsumura, Hiroshi Oji, Hironori Ofuchi, Masaharu Oshima

**Affiliations:** 1 Synchrotron Radiation Research Organization, The University of Tokyo, 7-3-1 Hongo, Bunkyo-ku, Tokyo 113-8656 Japan; 2 Institute for Solid State Physics, The University of Tokyo, 1-1-1 Kouto, Sayo-cho, Hyogo 679-5198 Japan; 3 Toyota Central R&D Labs., Inc, 41-1 Yokomichi, Nagakute, Aichi 480-1192 Japan; 4 Toyota Motor Corp., 1200 Mishuku, Susono, Shizuoka 410-1193 Japan; 5 Synchrotron Radiation Research Center, National Institutes for Quantum and Radiological Science and Technology, 1-1-1 Kouto, Sayo, Hyogo 679-5148 Japan; 6 Japan Atomic Energy Agency, SPring-8, 1-1-1 Kouto, Sayo, Hyogo 679-5148 Japan; 7 Japan Synchrotron Radiation Research Institute, 1-1-1 Kouto, Sayo-cho, Hyogo 679-5198 Japan; 8 Graduate School of Pure and Applied Science, University of Tsukuba, 1-1-1 Tennodai, Tsukuba, 305-8571 Japan; 9 Tokyo Institute of Technology, 2-12-1 Ookayama, Meguro-ku, Tokyo 152-8552 Japan

## Abstract

*In situ* high energy resolution fluorescence detection X-ray absorption spectroscopy (HERFD-XAS) was used to systematically evaluate interactions of H_2_O and O_2_ adsorbed on Pt and Pt_3_Co nanoparticle catalysts in different particle sizes. The systematic increase in oxidation due to adsorption of different species (H_2_O adsorption <O_2_ adsorption <O_2_ + H_2_O coadsorption) suggests that cooperative behavior between O_2_ and H_2_O adsorptions is responsible for the overpotential induced by hydrated species in fuel cells. From the alloying and particle size effects, it is found that both strength of O_2_/H_2_O adsorption and their cooperative effect upon coadsorption are responsible for the specific activity of Pt catalysts.

## Introduction

In the past decade, polymer electrolyte fuel cells (PEFCs) have attracted increasing attention as highly efficient power sources^1, 2^. The bottleneck to improve the energy-conversion efficiency of the fuel cells is the presence of the high overpotential in the oxygen reduction reaction (ORR) process^3^. In order to reduce the overpotential, a number of cathode catalysts, which are responsible for the ORR process, have been developed. Among them, Pt-based nanoparticles exhibit the best ORR performance and still dominate the fuel cell market. However, it is pointed out that interaction between the cathode catalysts and hydrated intermediate species causes overpotential during the ORR process^4^. Therefore, it is quite important to characterize the interaction of the hydrated species with catalysts based on the electronic structures.

According to the *d*-band center theory^5, 6^ the energy position of the center of the Pt 5*d* projected density of states (*d*-pDOS) is well correlated with the binding energy of adsorbates. Different adsorbates should have different correlations due to selective interaction of Pt *d*-pDOS with the valence orbitals of the adsorbates. Upon the adsorption, the Pt *d*-pDOS splits into bonding and antibonding states where the energy position of the antibonding states just above the Fermi level depends on the interaction with the adsorbates. Pt *L*-edge X-ray absorption fine structure (XAFS) is a relevant and the most frequently used method to probe the antibonding states. Based on the dipole selection rule the empty *d*-pDOS can be predominantly probed and the peak intensity of the absorption edge (white line) reflects the number of holes in the *d*-band^7, 8^, which has successfully been applied to explore the electronic structure of catalysts under various reaction conditions. However, differences in the XAFS spectra due to the presence of various adsorbates are relatively small, and it is difficult to discriminate differences in the electronic structure by conventional detection methods such as transmission and total fluorescence yields because fine structures in the spectra are smeared out by the lifetime broadening of the Pt 2*p*_3/2_ core hole. In contrast, novel high energy resolution fluorescence detection X-ray absorption spectroscopy (HERFD-XAS)^9–11^ enables us to extract a core-hole lifetime-broadening-reduced high resolution X-ray absorption spectrum by monitoring the monochromatized fluorescence line (such as Pt *L*_*α*1_) that is broadened only by the lifetime of the Pt 3*d*_5/2_ (~2.4 eV), not by that of the Pt 2*p*_3/2_ (~5.2 eV)^11^ core hole.

In this paper, *in situ* Pt *L*_3_ edge HERFD-XAS was performed for Pt and Pt_3_Co nanoparticles in different sizes under various gas conditions. Thanks to the high energy resolution, the interaction of O_2_ and H_2_O with the Pt surface and the (cooperative) effect of O_2_ and H_2_O coadsorption is clarified. Based on these results the origin of the overpotential by hydrated species will be discussed.

## Results and Discussion

**Figure 1:**
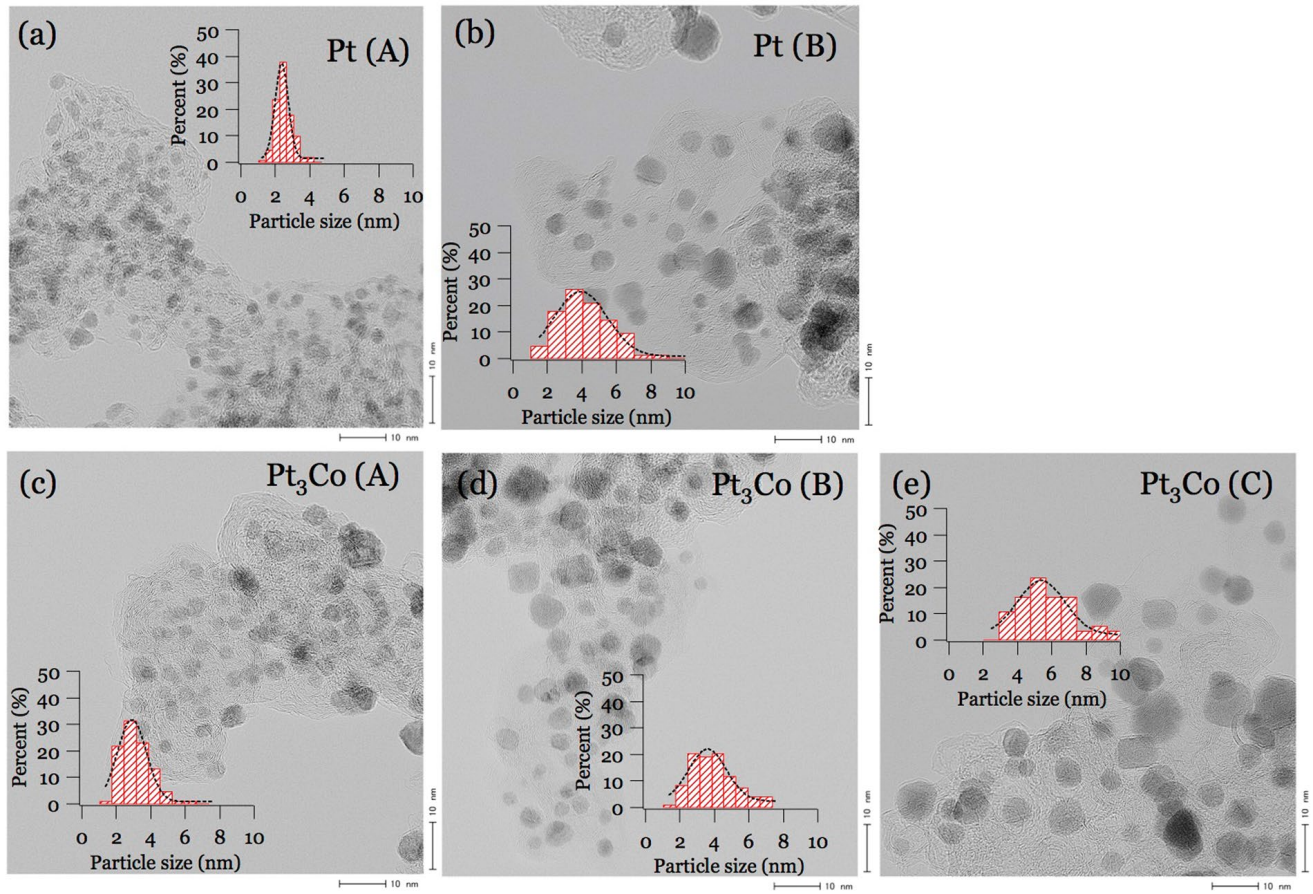
Typical TEM images of (**a**) Pt(A), (**b**) Pt(B), (**c**) Pt_3_Co(A), (**d**) Pt_3_Co(B), and (**e**) Pt_3_Co(C) samples. The inserts show the particle size distributions from TEM images.

**Figure 2:**
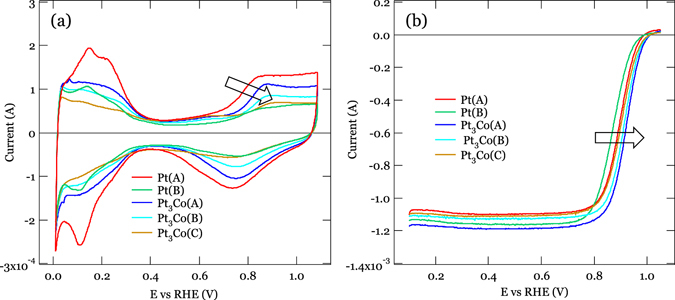
The electrochemical performance measurements. (**a**) Cyclic voltammetry of samples in this work. (**b**) The oxygen reduction curve by linear sweep voltammetry. The arrows marked the trends in cyclic voltammetry and oxygen reduction curve changes against different samples.

Pt(A) and (B) as well as Pt_3_Co (A), (B), (C) nanoparticles as specified in Table 1, were loaded on a black carbon and characterized by TEM, electrochemical measurement and XRD as presented in Figs 1 and 2 and S1, respectively. The evaluated particle sizes and size distributions are presented in Table 1. As shown in Fig. 1, the particle sizes measured by XRD are almost the same as estimated from the TEM images, indicating that fine crystalline particles are formed. The cyclic voltammetry and oxygen reduction curves are shown in Fig. 2. The Pt_3_Co samples have better electrochemical performance than Pt nanoparticles in their high Pt oxidization voltage and relatively high half wave values.

**Table 1:** Sample specifications (Pt and Pt_3_Co nanoparticles loaded on a carbon support).

Sample name	Particle size (nm)		Pt (wt %)	Surface area ratio (m^2^/gPt)
	XRD (error bar)	TEM (distribution)		CO stripping
Pt(A)	2.1 ± 0.2	2.4 ± 0.5	46.6	160.3
Pt(B)	4.4 ± 0.3	4.3 ± 1.7	52.7	97.7
Pt_3_Co(A)	3.3 ± 0.2	3.2 ± 1.0	48	92.6
Pt_3_Co(B)	4.1 ± 0.2	4.0 ± 1.4	48.7	85.9
Pt_3_Co(C)	4.9 ± 0.3	5.8 ± 1.5	49	65.3

**Figure 3:**
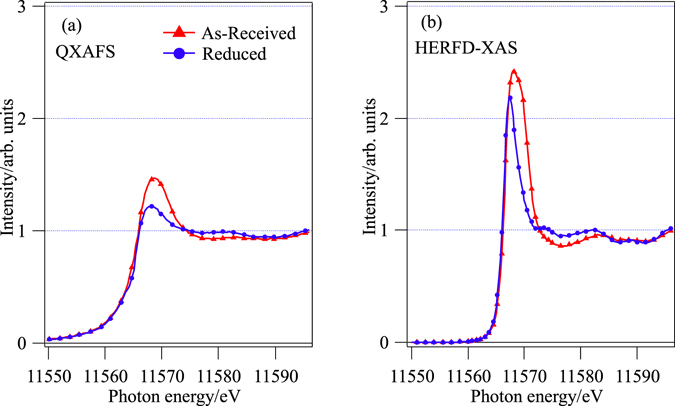
Pt *L*_3_ edge XAS of Pt(A) with (**a**) QXAFS and (**b**) HERFD-XAS methods under as-received and reduced conditions. More features are distinguished in the high resolution HERFD-XAS spectra than QXAFS.

The Pt *L*_3_ edge XAS spectra of the smallest Pt nanoparticle catalyst: Pt(A) measured with QXAFS and HERFD-XAS methods for as-received and reduced conditions are shown in Fig. 3(a) and (b), respectively. They are normalized by the intensity at isosbestic point around 11594 eV. The white line intensity and its profile are more clearly distinguished in the HERFD-XAS spectra for different chemical states than those of the QXAFS spectra. Upon reduction the white line intensity is reduced and the peak energy is red-shifted as expected. We will not go into detail the interpretation of the XAS profile of the as-received sample since complicated oxidation process might have occur during the sample fabrication and air exposure before the experiment. As described in Fig. S2, the QXAFS spectra were explicitly reproduced by the HERFD-XAS spectra convoluted with the lifetime broadening of Pt 2*p*_3/2_ (5.2 eV) after deconvolution with Pt 3*d*_5/2_ (2.4 eV) core levels.

**Figure 4:**
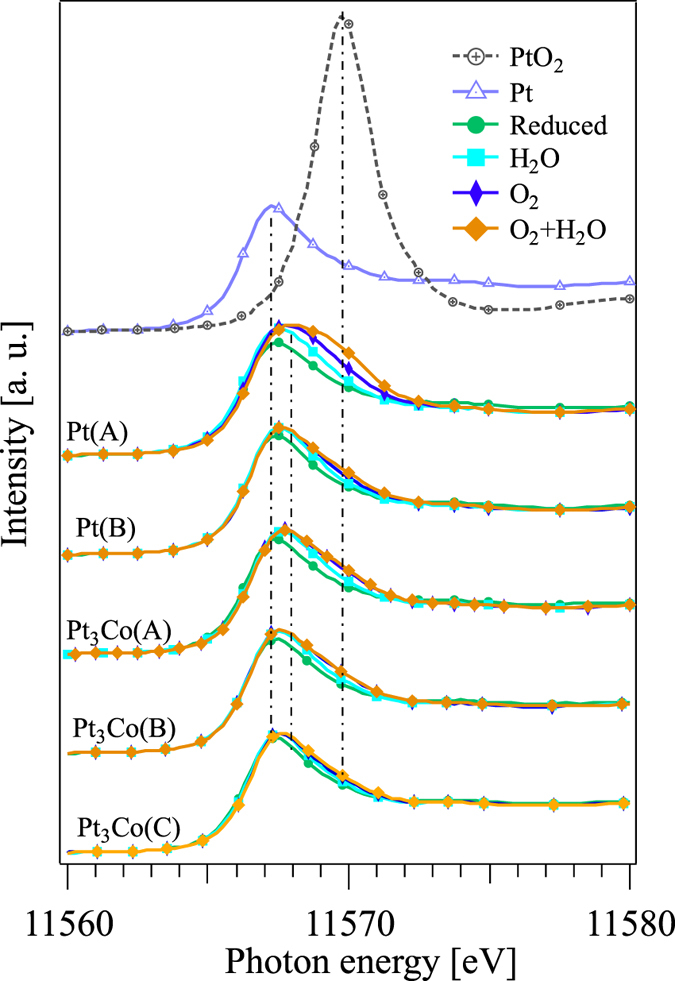
*In situ* HERFD-XAS spectra of Pt and Pt_3_Co nanoparticles under reduced condition, O_2_, H_2_O, as well as O_2_ + H_2_O adsorptions for Pt(A), Pt(B), Pt_3_Co(A), Pt_3_Co(B), Pt_3_Co(C) samples from top to the bottom. HERFD-XAS spectra of the Pt foil and PtO_2_ powder were also shown as standards of fully reduced and oxidized conditions. The vertical dotted-broken lines are drawn to the peak positions of Pt foil, PtO_2_, and Pt(A) under O_2_ + H_2_O adsorption.

**Figure 5:**
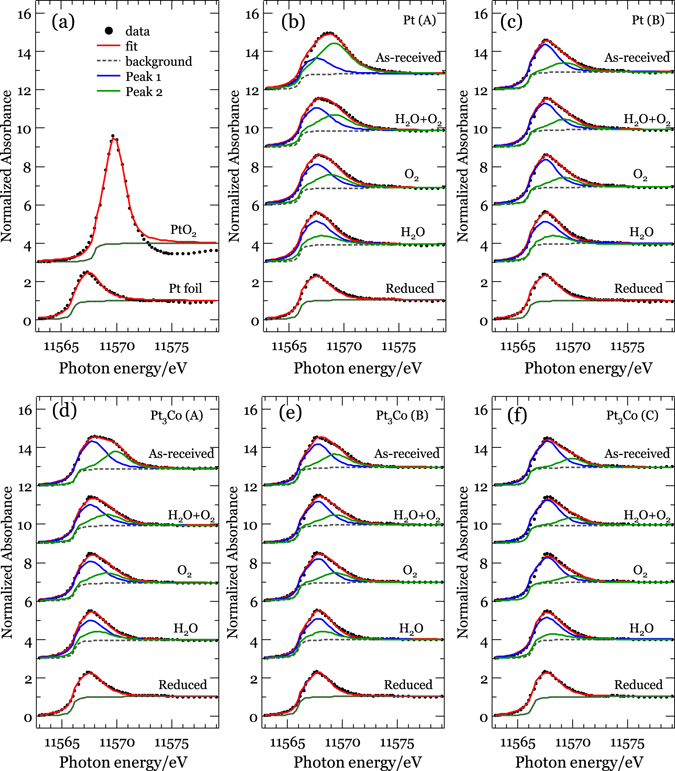
Least-squares fits by two peaks for all HERFD-XAS spectra from Fig. 4. (**a**) Pt foil and PtO_2_ powder, (**b**) Pt(A), (**c**) Pt(B), (**d**) Pt_3_Co(A), (**e**) Pt_3_Co(B) and (**f**) Pt_3_Co(C) samples, respectively.

**Figure 6:**
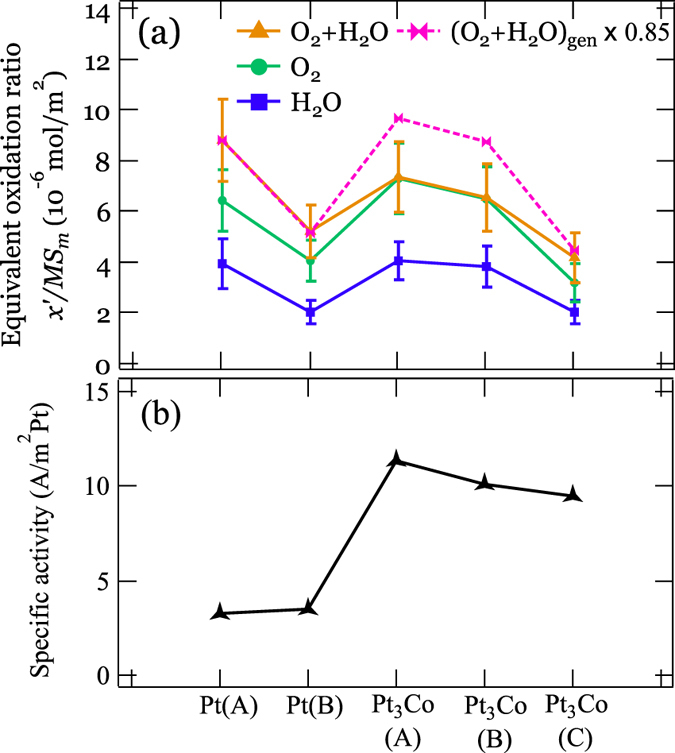
The comparison of (**a**) Equivalent oxidation ratios in different *in situ* conditions and (**b**) Specific activities for all the samples. The curves in (**a**) show the trends of H_2_O, O_2_ and O_2_ + H_2_O adsorptions against different samples. The 1:1 sum of the spectra for O_2_ and H_2_O adsorption with a reduction factor of 0.85 were presented for references comparing to the equivalent oxidation ratio for the O_2_ + H_2_O coadsorption.

Figure 4 shows the results for *in situ* HERFD-XAS spectra of Pt and Pt_3_Co nanoparticles with different particle sizes under reduced, O_2_, H_2_O, and O_2_ + H_2_O adsorption conditions. For all samples, the white line intensity changes depending on the adsorption conditions. According to the procedure described in section 3 of Supplementary Information, the obtained HERFD-XAS spectra were decomposed into oxidized (PtO_2_ as a reference) and reduced (Pt foil as a reference) components by least-square fits as shown in Fig. 5. The ratio of the peak area intensity of the fitted two components divided by the Pt catalytic surface area estimated from CO stripping^12^ is shown in Fig. 6(a) for the Pt and Pt_3_Co catalysts, which we denote “equivalent oxidation ratio”. The equivalent oxidation ratio is proportional to the surface coverage assuming similar charge transfer among the catalysts (see Section 3 of Supplementary Information). H_2_O adsorption shows the lowest equivalent oxidation ratio, which is consistent with the weak bonding of H_2_O on Pt surfaces reported by Cui *et al*.^13^ and Zimbitas *et al*.^14^. As shown in Fig. 6(a), coadsorption of O_2_ and H_2_O, denoted as O_2_ + H_2_O, exhibits the highest equivalent oxidation ratio, regardless of the sequence of adsorption (as shown in Fig. S3 for O_2_ + H_2_O, H_2_O + O_2_ as well as O_2_ and H_2_O coadsorptions). We show here that a simple model only taking either O_2_ or H_2_O adsorption into account cannot explain the specific activity of the catalysts. (O_2_ + H_2_O)_gen_ in Fig. 6(a) is the 1:1 sum of the spectra for O_2_ (green) and H_2_O (purple) adsorption, which represents a virtual case where both O_2_ and H_2_O molecules adsorb independently on the Pt surface and both have the same oxidation effect on the Pt atom (see Section 3 of the Supplementary Information). Practically O_2_ or H_2_O molecules covering the surface should reduce the effective adsorption sites for H_2_O or O_2_, respectively. Considering the covering effect the equivalent oxidation ratio of (O_2_ + H_2_O)_gen_ should be higher than that of O_2_ + H_2_O. Here we introduce a reduction factor 0.85 as the lowest limit where the equivalent oxidation ratio of (O_2_ + H_2_O)_gen_ overlaps with O_2_ + H_2_O for Pt(A), Pt(B), and Pt_3_Co(C) catalysts. It is noted that enhancement of oxidation by coadsorption (cooperative effect) is significantly suppressed or even negligible for O_2_ + H_2_O coadsorption on Pt_3_Co(A) and Pt_3_Co(B) catalysts, which implies the presence of additional exclusion effect between O_2_ and H_2_O adsorption other than the surface covering effect.

Here we discuss the relationship between the cooperative effect and the activity of the catalysts. Figure 6(b) demonstrates specific activity of all the catalysts obtained by the current density normalized to the electrochemical surface area. Details of the measurement are described in the subsection “The electrochemical performance measurement” of Methods. Higher specific activity is demonstrated for Pt_3_Co than Pt, having difference in the activity among Pt_3_Co with different particle sizes. As shown in Fig. 6(a) and (b), the reduced or suppressed oxidation (or absence of the cooperative effect) for Pt_3_Co(A) and Pt_3_Co(B) catalysts has a significant effect on the specific activity, while it is intriguing to note that the equivalent oxidation ratio of O_2_ or H_2_O molecule does not have simple correlation with the specific activity. These results could explain how the hydrated species cause overpotential, suggesting that the elimination of the cooperative effect on the adsorption of O_2_ and H_2_O molecules can be a clue to improve the ORR performance of PEFCs.

The results obtained in this study are not contradictory to the conventional *d*-band center theory. As revealed by Xu *et al*.^15^, there is a linear relationship between atomic oxygen binding energy and the oxygen dissociation barrier of the transition metals and alloys. Following the Sabatier principle^1, 2, 16^ the more strongly a molecule is bonded to a material, the more effectively dissociation of the molecule occurs, resulting in a stable monoatomic oxygen adsorption for both O_2_ and H_2_O adsorption, and causing high overpotential or limiting the successive ORR reaction. Therefore, the well-known volcano-type plot^17, 18^ can explain various experimental results. In this study, however, in addition to the *d*-band center theory, we suggest that the cooperative effect between O_2_ and H_2_O adsorption also contribute to the overpotential. It is also worth to mention about the particle size effect. For both Pt and Pt_3_Co, H_2_O and O_2_ adsorption as well as their coadsorption are decreased with increasing the particle size. For Pt, the less coadsorption on the larger Pt(B) than on Pt(A) contributes to the slightly higher specific activity. For Pt_3_Co having less cooperative effect than Pt, on the contrary, the less coadsorption on the largest Pt_3_Co(C) does not result in the increase of the specific activity but the rapid decrease of O_2_ adsorption upon the increase of the particle size would be responsible for the decrease in the specific activity. Therefore, the different trend against the particle size for Pt and Pt_3_Co should be a matter of competition between the Sabatier principle and the cooperative effect. Further study including high resolution *operando* XAFS of real PEFC will be helpful to reveal the cooperative effect. In this study the relationship between the cooperative effect and the shape or the morphology of the nanoparticles is not discussed, which will also be an important issue to discuss the real PEFC catalysts in the near future.

In summary, by using HERFD-XAS, we have investigated the variation in the electronic structure of a series of Pt and Pt_3_Co nanoparticles upon O_2_ and H_2_O adsorption. The high energy resolution is definitely important for the precise peak fitting of the white line depending on the adsorbed molecules, which is essential to explain why the hydrated species increase the overpotential and decrease the energy conversion efficiency in PEFCs. Among the samples in this study, it is found that the strength of oxidation by O_2_ or H_2_O adsorption does not have simple correlation with the specific activity, while the suppressed oxidation by the coadsorption of O_2_ and H_2_O for the Pt_3_Co(A) and Pt_3_Co(B) catalysts has correlation with the high specific activity. Taking both alloying and particle size effects into account it is expected that both the strength of molecular adsorption (Sabatier principle) and the cooperative effect among those adsorptions, which is the origin of the overpotential by hydrated species, contribute to the specific activity of Pt catalysts.

## Methods

### Sample characterization

Catalysts used in this study are commercially available from Tanaka Kikinzoku Kogyo (TKK) Co., Ltd., Japan and listed in Table 1. The particle size and its distribution of each sample were estimated by TEM images and the Scherrer equation^19^ using the (220) XRD peak (Fig. S1) fine scanned with Rigaku SmartLab XRD spectrometer. In this study Pt(B) and Pt_3_Co(B) having similar particle sizes were chosen in order to extract the alloying effect, while the other three samples were chosen in order to extract the particle size effect. We limited the size of the nanoparticles less than 6 nm in order to contrast the surface signal for the bulk sensitive XAS.

Spherical aberration-corrected transmission electron microscopy (TEM) images were obtained on a JEOL JEM-ARM200F equipped with a guaranteed point resolution of 0.12 nm at 200 K. Before microscopy examination, the samples were ultrasonically dispersed in ethanol, and then the solution was dropped on a copper TEM grid. The particle size and its distribution were obtained by analyzing ~100 Pt nanoparticles from the TEM images.

The chemical composition of the samples were checked by hard X-ray photoelectron spectroscopy (HAXPES) using 7939 eV incident photons monochromatized with Si (111) double-crystal and Si (444) channel-cut monochromators^20, 21^ with a VG Scienta R4000 hemispherical electron analyzer at the undulator beamline BL46XU of SPring-8. The survey spectra of Pt(A), Pt_3_Co(B) as well as Pt and Pt_3_Co bulk samples are shown in Fig. S4. The peak intensity ratio of Pt 4 *f* and Co 2*p* core levels in Pt_3_Co nanoparticles are the same as that in the bulk Pt_3_Co, which indicate that Pt:Co atomic ratio in nanoparticles is around 3:1, since HAXPES can probe enough bulk information up to ~20 nm in the Pt_3_Co bulk sample.

### The electrochemical performance measurements

The electrochemical measurements were carried out with a rotating disc electrode (RDE, Hokuto Denko) in which a Pt wire was used as the counter electrode and a normal hydrogen electrode was used as the reference electrode. The catalyst ink was prepared by blending the 30 mg of catalyst powder in isopropyl alcohol (IPA, 7.5 ml) and Nafion solution (5 wt.%, 131 μl) and then 10 μl of the ink was deposited onto the 5 mm diameter glassy carbon RDE. For each electrochemical measurement, the electrodes were cycled 20 times between 0.05 and 1.00 V in Ar saturated solution, to obtain a relatively stable and clean surface at a scan rate of 100 mVs^−1^. The cyclic voltammetry (CV) scans within the potential range between 0.01 and 1.0 V at a sweeping rate of 50 mVs^−1^ under an Ar atmosphere. The electrochemical surface area (ECSA) was obtained by integrating the areas of H desorption between 0.05 and 0.40 V after the deduction of the double-layer region. The oxygen reduction current was calculated by linear sweep voltammetry with a scan rate of 10 mVs^−1^ and a rotational rate of 1600 rpm in a high-purity O_2_ saturated 0.1 M HClO_4_ aqueous solution at room temperature. For all catalysts in this work, the specific activity (SA) and mass activity (MA) were obtained by the current density normalized to the ECSA and Pt loading, respectively.

### *In situ* QXAFS and HERFD-XAS

*In situ* Pt *L*_3_-edge transmission mode quick X-ray absorption fine structure (QXAFS) measurement was carried out at SPring-8 (8 GeV, 100 mA), the industrial application bending magnet beamline II - BL14B2^22, 23^ of SPring-8, while *in situ* high-resolution fluorescence detection X-ray absorption (HERFD-XAS) measurements were carried out at the contract undulator beamline BL11XU of SPring-8. For the transmission QXAFS measurements at BL14B2, a single pair of Si (311) double-crystal monochromator were used to monochromatize X-ray beam. The beam was focused at the sample position using a Rh coated focusing mirror with the beam size of 5.0 mm in the horizontal direction and 0.5 mm in the vertical direction^23^ with a photon flux of ~10^10^ photons/sec and energy resolution ΔE/E of 10^−4^. For the HERFD-XAS measurements at BL11XU, the X-ray beam was monochromatized with a Si (111) double-crystal monochromator followed by a 2-bounce Si (400) channel-cut monochromator to further increase the energy resolution (~400 meV)^24, 25^. The emitted X-rays in the horizontal plane were analyzed by a Rowland-mount type emission spectrometer equipped with a spherically bent Si perfect crystal (R = 2 m) which was aligned in a backscattering geometry using (733) Bragg reflection to select the Pt *Lα*_1_ fluorescence line (9442 eV). The incident photon energy was calibrated by aligning the inflection point of the Pt *L*-edge XAS leading edge of the Pt foil.

The as-received powder samples mixed with BN powder were filled in a quartz tube (inner diameter *φ* 7 mm) with the thickness around 3 mm for *in situ* QXAFS measurements, while for *in situ* HERFD-XAS, samples were directly filled in a *φ* 7 mm stainless steel tube with the thickness around 1 mm. These samples were installed into *in situ* cells (see Figs S5 and S6). For one sample, the following set of experiments was carried out:

1. The sample is first reduced by 1 atm. 200 °C H_2_ (10%) +He (90%) gas flowing at 100 ml/min for one hour.
2. After the reduction, the sample is filled with 1 atm. O_2_ gas flowing at 100 ml/min at 30 °C for 10 mins.
3. The sample is reduced again, and 1 atm. fully humidified N_2_ gas flowing at 100 ml/min is supplied at 30 °C for 10 mins.
4. Steps 1–3 are repeated and then 1 atm. fully humidified O_2_ gas flowing at 100 ml/min is supplied at 30 °C for 10 mins.

The QXAFS and HERFD-XAS spectra were obtained for as-received samples and *in situ* condition after stabilizing each step of the above procedure. Coadsorption of O_2_ and H_2_O was examined under the following three conditions: (1) H_2_O (N_2_ as carrier gas) adsorption followed by O_2_ adsorption (H_2_O + O_2_), (2) O_2_ adsorption followed by H_2_O (N_2_ as carrier gas) adsorption (O_2_ + H_2_O), and (3) the above step 4 (O_2_ with H_2_O). The results provided the same intensity and position of the Pt *L*_3_ white line as shown in Fig. S3, which means that coadsorption takes place in the same manner regardless of the sequence of O_2_ and H_2_O adsorption. Therefore, we simply use the term O_2_ + H_2_O for coadsorption.

The QXAFS and HERFD-XAS spectra were analyzed using an XAS analysis program Demeter Athena with the Autobk program^26^. The quantitative analysis of QXAFS and HERFD-XAS was carried out by two methods for confirmation; one is a fitting by the linear combination of measured spectra for well-reduced Pt nanoparticle and for standard *β*-PtO_2_. The other is least square fits of the white line peaks using two peaks consisting of pseudo-Voigt (Gaussian−Lorentzian product) function followed by subtraction of an arctangent-type background. The height of the arctangent jump was tuned to match the absorption at energies around isosbestic point.

## Electronic supplementary material

Supplementary information
