# Supplementary material for: Wetting Induced Oxidation of Pt-based Nano Catalysts Revealed by In Situ High Energy Resolution X-ray Absorption Spectroscopy
Source: Sci Rep. 2017 May 3;7:1482. doi: 10.1038/s41598-017-00639-1 (PMC5431092; doi:10.1038/s41598-017-00639-1)
Supplement: Supplementary file 1 — Supplementary information [file 41598_2017_639_MOESM1_ESM.pdf]

Supplementary information

Wetting Induced Oxidation of Pt-based Nano Catalysts Revealed by *In Situ* High Energy Resolution X-ray Absorption Spectroscopy

Yi-Tao Cui[1,*], Yoshihisa Harada[1,2], Hideharu Niwa[1,2,8], Tatsuya Hatanaka[3], Naoki Nakamura[4], Masaki Ando[4], Toshihiko Yoshida[4,9], Kenji Ishii[5], Daiju Matsumura[6], Hiroshi Oji[7], Hironori Ofuchi[7], Masaharu Oshima[1,†]

[1]Synchrotron Radiation Research Organization, The University of Tokyo, 7-3-1 Hongo, Bunkyo-ku, Tokyo, 113-8656, Japan

[2]Institute for Solid State Physics, The University of Tokyo, 1-1-1 Kouto, Sayo-cho, Hyogo 679-5198, Japan

[3]Toyota Central R&D Labs., Inc., 41-1 Yokomichi, Nagakute, Aichi, 480-1192, Japan

[4]Toyota Motor Corp. 1200 Mishuku, Susono, Shizuoka 410-1193, Japan

[5]Synchrotron Radiation Research Center, National Institutes for Quantum and Radiological Science and Technology, 1-1-1 Kouto, Sayo, Hyogo 679-5148, Japan

[6]Japan Atomic Energy Agency, SPring-8, 1-1-1 Kouto, Sayo, Hyogo 679-5148, Japan

[7]Japan Synchrotron Radiation Research Institute, 1-1-1 Kouto, Sayo, Hyogo 679-5198, Japan

[8]Present address: Graduate School of Pure and Applied Science, University of Tsukuba, 1-1-1 Tennodai, Tsukuba 305-8571, Japan.

[9]Present address: Tokyo Institute of Technology, 2-12-1 Ookayama, Meguro-ku, Tokyo 152-8552, Japan

Corresponding authors: *yitaocui@issp.u-tokyo.ac.jp, † oshima@sr.t.u-tokyo.ac.jp

1. Estimation of the particle size by using the Pt XRD pattern

The mean particle size of each sample (as shown in Table I) was estimated by the Scherrer equation[1]:

$$D = \frac{K\lambda}{B_{2\theta}\cos\theta} \quad (1)$$

where the error bar of the particle size can be described as:

$$\Delta D = \left|\frac{\partial D}{\partial B_{2\theta}}\right|\Delta B_{2\theta} + \left|\frac{\partial D}{\partial K}\right|\Delta K + \left|\frac{\partial D}{\partial \theta}\right|\Delta\theta \quad (2)$$

where D is the mean size of the ordered (crystalline) domains, maybe smaller or equal to the particle size, λ is the X-ray wave length, K is a dimensionless shape factor called Scherrer constant, θ is the Bragg angle. $B_{2\theta}$ is the line broadening estimated by the full width at half maximum of the Bragg peak after subtracting the instrumental line broadening. The (220) XRD peak (inset of Fig. S1) fine scanned with Rigaku SmartLab XRD spectrometer was used for evaluation of the particle sizes based on Eq. (1).

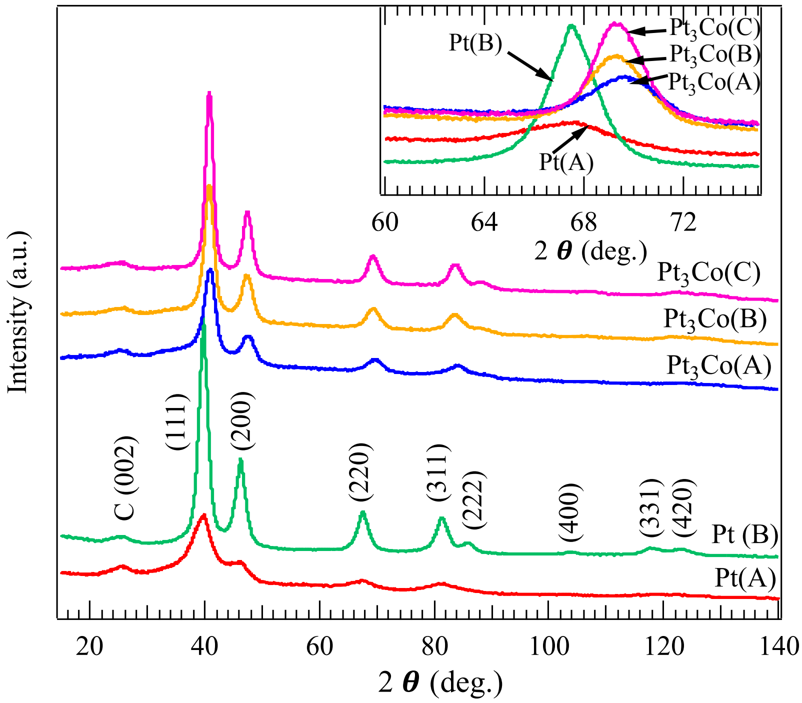

Fig. S1 XRD pattern of as-received samples for Pt(A), Pt(B), Pt_3Co(A), Pt_3Co(B), Pt_3Co(C). The inset shows the expanded view of the XRD pattern around the (220) peak.

2. The relationship of HEFRD-XAS and transmission mode XANES.

Convolution of the HERFD-XAS spectrum of Pt(A) with a differential lifetime broadening between Pt 2$p_{3/2}$ (~5.2 eV) and Pt 3$d_{5/2}$ (~2.4 eV) [2], namely 4.6 eV ($\sqrt{(5.2^2-2.4^2)}$), exactly reproduces the XAS spectrum measured by a transmission method as shown in Fig. S2.

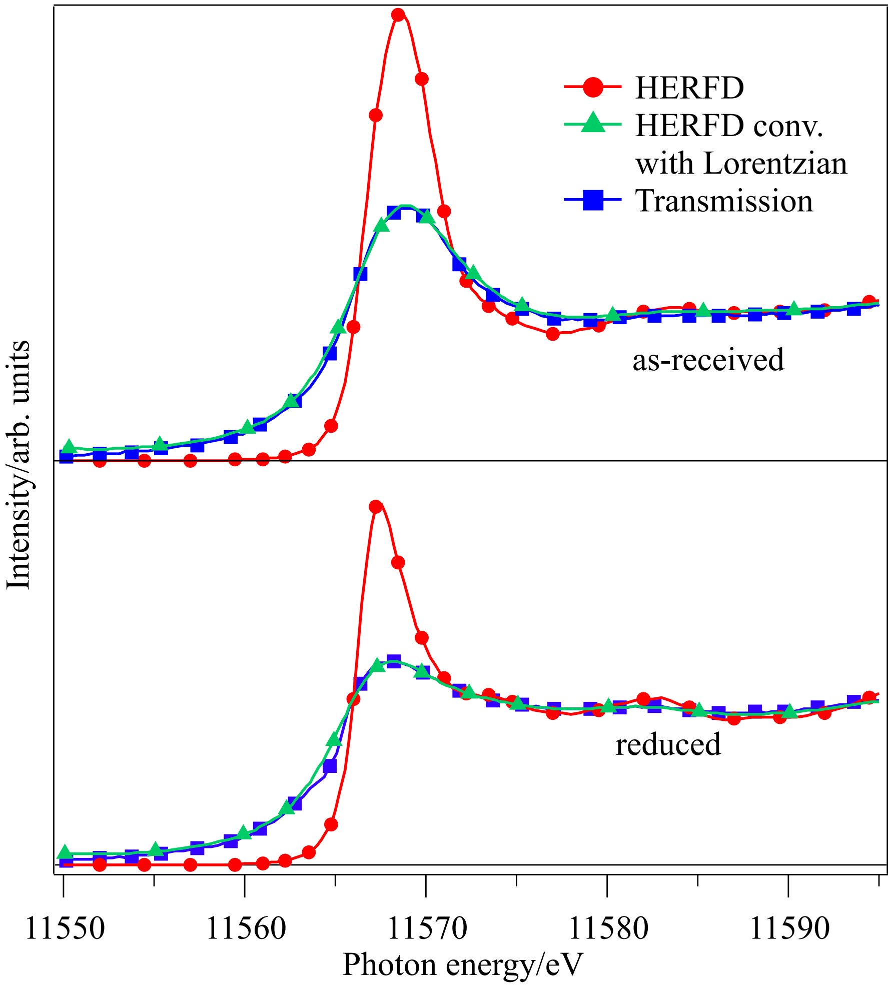

Fig. S2 Pt L_3 edge XANES of Pt(A) with transmission detection and HERFD as well as that of convoluted HERFD spectrum.

3. Peak fitting of the XANES and HERFD-XAS spectra in Fig. 5 and the meaning of $\frac{x'}{MS_m}$ in Fig. 6

The basic knowledge about the linear relationship of Pt L_3 white line intensity and Pt *d* electron vacancies was reported by Lytle et al.[3] and Horsley[4]. Our peak area fitting of the Pt L_3 XANESs spectra for standard Pt foil and PtO_2 samples is also based on the solution described by Horsley[4].
Considering the relationship between HERFD-XAS and transmission mode XANES shown in Fig. S2, one can use the same fitting to analyse the HERFD-XAS spectra and obtain strength of hybridization between adsorbates and Pt atoms more accurately.

When the white line area of Pt and PtO_2 are denoted as μ_{Pt} and μ_{PtO2}, the change in the white line area can be written as $\frac{\mu_{PtO2}-\mu_{Pt}}{\delta_{PtO2}}\cdot\delta$, where δ_{PtO2} is the change in the *d* valence from Pt to PtO_2, which is around 1.2 electrons per Pt atom[4] and δ is the number of charge transferred electrons per Pt atom.
The white line area (μ) for a certain adsorption to the Pt atom with the ratio x and the charge transfer δ can be written as:

$$\mu = \mu_{Pt} + x\cdot\frac{\mu_{PtO2}-\mu_{Pt}}{\delta_{PtO2}}\cdot\delta \tag{3}$$

Then,

$$\frac{\mu-\mu_{Pt}}{\frac{\mu_{PtO2}-\mu_{Pt}}{\delta_{PtO2}}} = x\cdot\delta \text{ or } \frac{\mu-\mu_{Pt}}{\mu_{PtO2}-\mu_{Pt}} = x\cdot\frac{\delta}{\delta_{PtO2}} = x' \tag{4}$$

The physical meaning of x' is the effective ratio of PtO_2 in the total number of Pt atoms and can be measured experimentally.

Given Θ the number of surface atom adsorbed by adsorbates, S the surface area, the surface coverage (mol/m^2) of adsorbates σ can be written as:

$$\sigma = \frac{\Theta}{S} = \frac{\Theta}{N_T}\cdot\frac{N_T}{S} = x\cdot\frac{N_T}{S} \tag{5}$$

Here, N_T is the number of Pt atoms with mass of m and can be rewritten using the Pt Molar mass M as:

$$N_T = \frac{m}{M} \tag{6}$$

Therefore,

$$\sigma = x \cdot \frac{\frac{m}{M}}{S} = \frac{1}{M \cdot \frac{S}{m}} \cdot x \tag{7}$$

Here, the surface area per gram of Pt (m^2/g) is defined as $S_m = \frac{S}{m}$, then,

$$\sigma = \frac{1}{MS_m} \cdot x \tag{8}$$

$$x = x' \cdot \frac{\delta_{PtO2}}{\delta} = \frac{\mu - \mu_{Pt}}{\mu_{PtO2} - \mu_{Pt}} \cdot \frac{\delta_{PtO2}}{\delta} \tag{9}$$

$$\sigma = x' \cdot \frac{\delta_{PtO2}}{\delta} \cdot \frac{1}{MS_m} \tag{10}$$

$$\frac{x'}{MS_m} = \sigma \cdot \frac{\delta}{\delta_{PtO2}} = \frac{\mu - \mu_{Pt}}{\mu_{PtO2} - \mu_{Pt}} \cdot \frac{1}{MS_m} \tag{11}$$

Therefore, we can use the measurable value $\frac{x'}{MS_m}$ instead of $\sigma \cdot \frac{\delta}{\delta_{PtO2}}$ since x' can be measured by XAFS or HERFD-XAS, and S can be measured by gas adsorption or electrochemical methods or evaluated by XRD or TEM.

Using the above equation one can quantitatively compare different adsorption states by *d* electron vacancies per surface Pt atom, since transmission mode XANES or fluorescence HERFD-XAS can equally detect signals from the surface and the core of the Pt nanoparticles.

Here the meaning of $\frac{x'}{MS_m}$ is summarized;

- Supposing a linear relationship of Pt 5*d* electron vacancy and Pt L_3 white line intensity, x' will be proportional to the number of Pt 5*d* electron vacancy. If either δ or x is known, the other can be solved.
- For the same adsorbate on different samples, supposing δ is the same, the $\frac{x'}{MS_m}$ value can be used to quantify σ.
- For different adsorbates on the same surface, if some models are proposed (to give the value of σ or δ) the $\frac{x'}{MS_m}$ value can be used to evaluate the ORR activity.

- S_m can be evaluated not only by CO-stripping voltammetry and hydrogen under potential deposition method but also by the diameter of nanoparticles obtained by XRD or STEM[5]. In this work, S_m was evaluated by CO-stripping voltammetry (TKK Co., Ltd.)

$$S_m = \frac{Q_{\mathrm{CO}}}{m \cdot C} \qquad (12)$$

where, Q_{CO} is the charge correlated with CO-stripping, and a constant of C = 4.20 C/m^2 is commonly used for the unit charge per surface area with CO-stripping on Pt or Pt_3Co surface as described in Ref. 5.

The error bar of $\frac{x'}{MS_m}$ can be described as:

$$\Delta\left(\frac{x'}{MS_m}\right) = \left|\frac{\partial \frac{x'}{MS_m}}{\partial \mu}\right| \Delta\mu + \left|\frac{\partial \frac{x'}{MS_m}}{\partial S_m}\right| \Delta S_m \qquad (13)$$

Usually, the system error of $\Delta S_m / S_m$ was varied from 11% to 17% as reported in Ref. 5. We applied 15% in this work.

By using the above method, the data shown in Fig. 6 were generated.

4. Comparison of the Pt L_3 edge XANES profiles of Pt(A) for different sequence of O_2 and H_2O adsorption

To check the effects of O_2 and H_2O adsorption on clean Pt surfaces, several sequence of adsorptions are examined as described in figure captions.

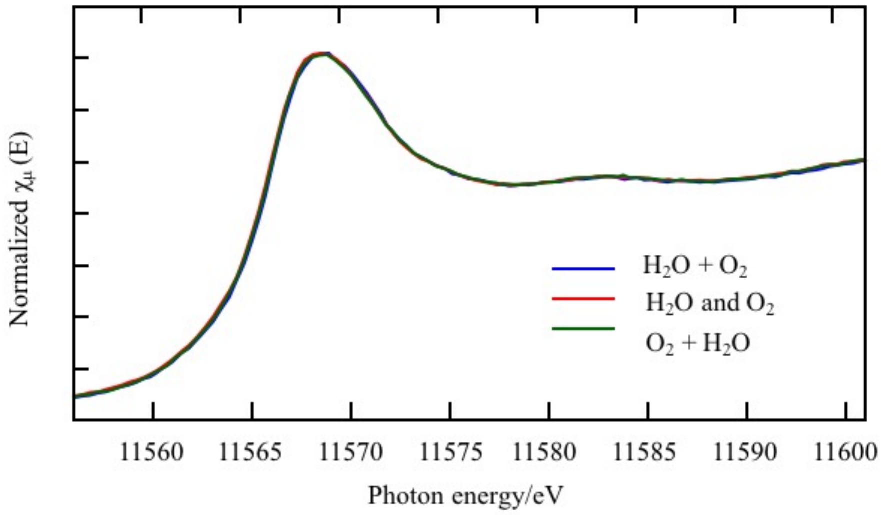

Fig. S3 Different sequence of O_2 and H_2O adsorptions on Pt(A). H_2O+O_2 means H_2O (N_2 as carrier gas) adsorption and then O_2 adsorption, O_2+H_2O means O_2 adsorption and then H_2O (N_2 as carrier gas) adsorption, O_2 and H_2O means O_2 as carrier gas of H_2O.

5. Samples evaluated by hard X-ray photoelectron spectroscopy (HAXPES)

For the HAXPES measurements the samples were fixed to Cu holders by carbon conducting tapes. The energy position of the Au $4f_{7/2}$ peak was used for energy calibration. The take-off-angle (TOA) was fixed to 80°. A total energy resolution of 230 meV was evaluated by the energy position of the Au Fermi edge at room temperature.

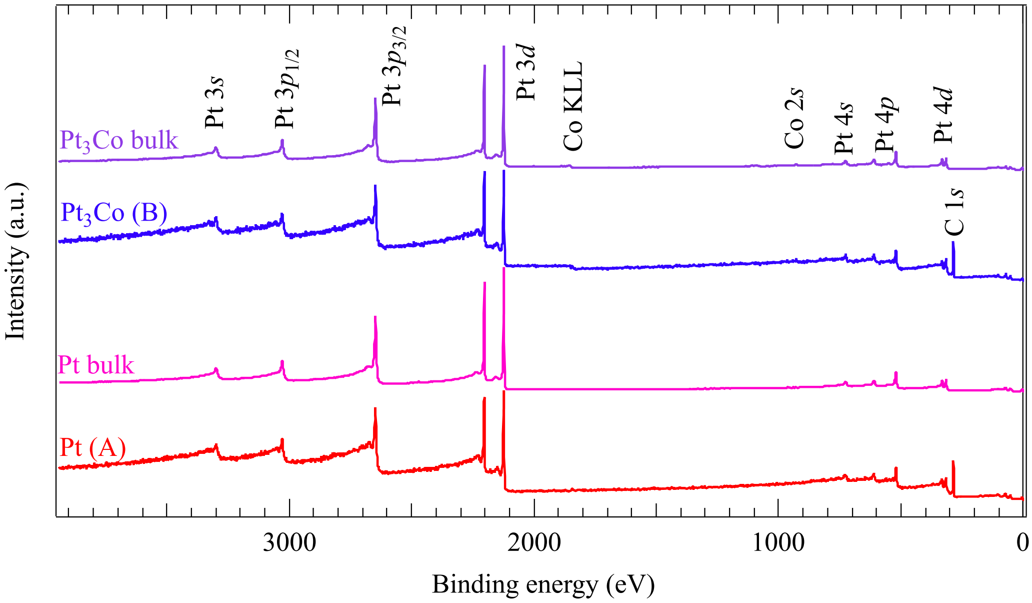

Fig. S4 HAXPES spectra of Pt(A), Pt_3Co(B) as well as Pt and Pt_3Co bulk samples.

6. **Setup of *in situ* XAFS experiment and the *in situ* cells.**

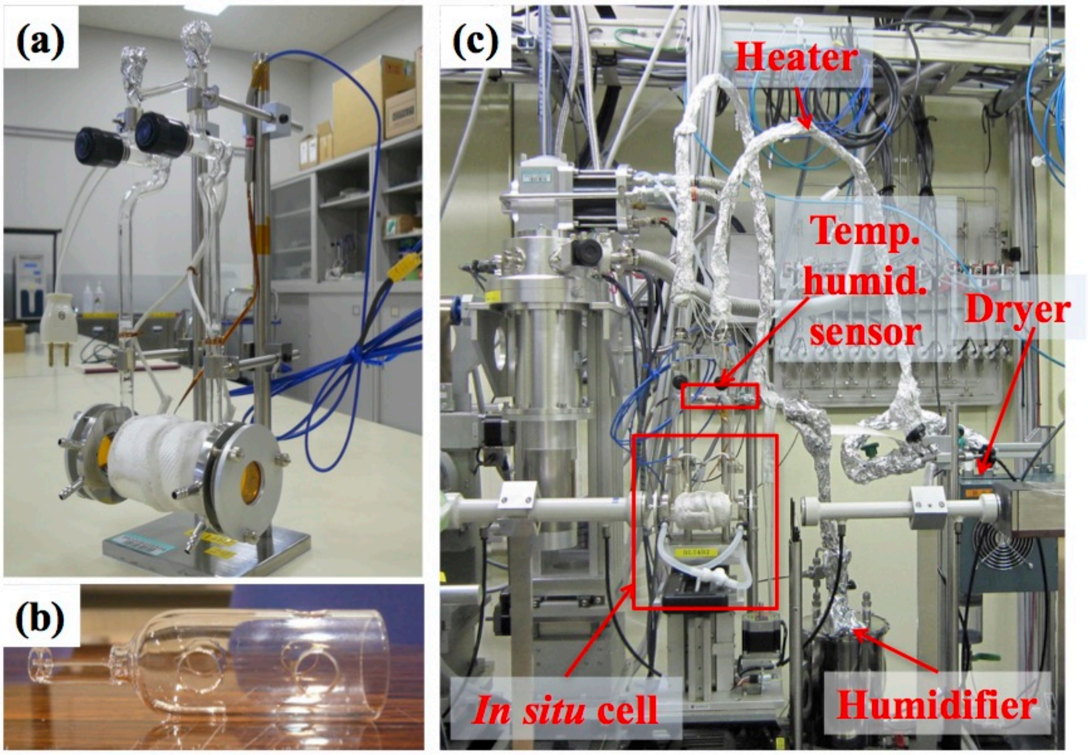

Fig. S5 Experimental setup for *in situ* XAFS experiment. (a) *In situ* cell. (b) Sample holder. (c) Setup of the *in situ* cell during XAFS experiment.

7. Setup of *in situ* HERFD-XAS experiment and the *in situ* cell.

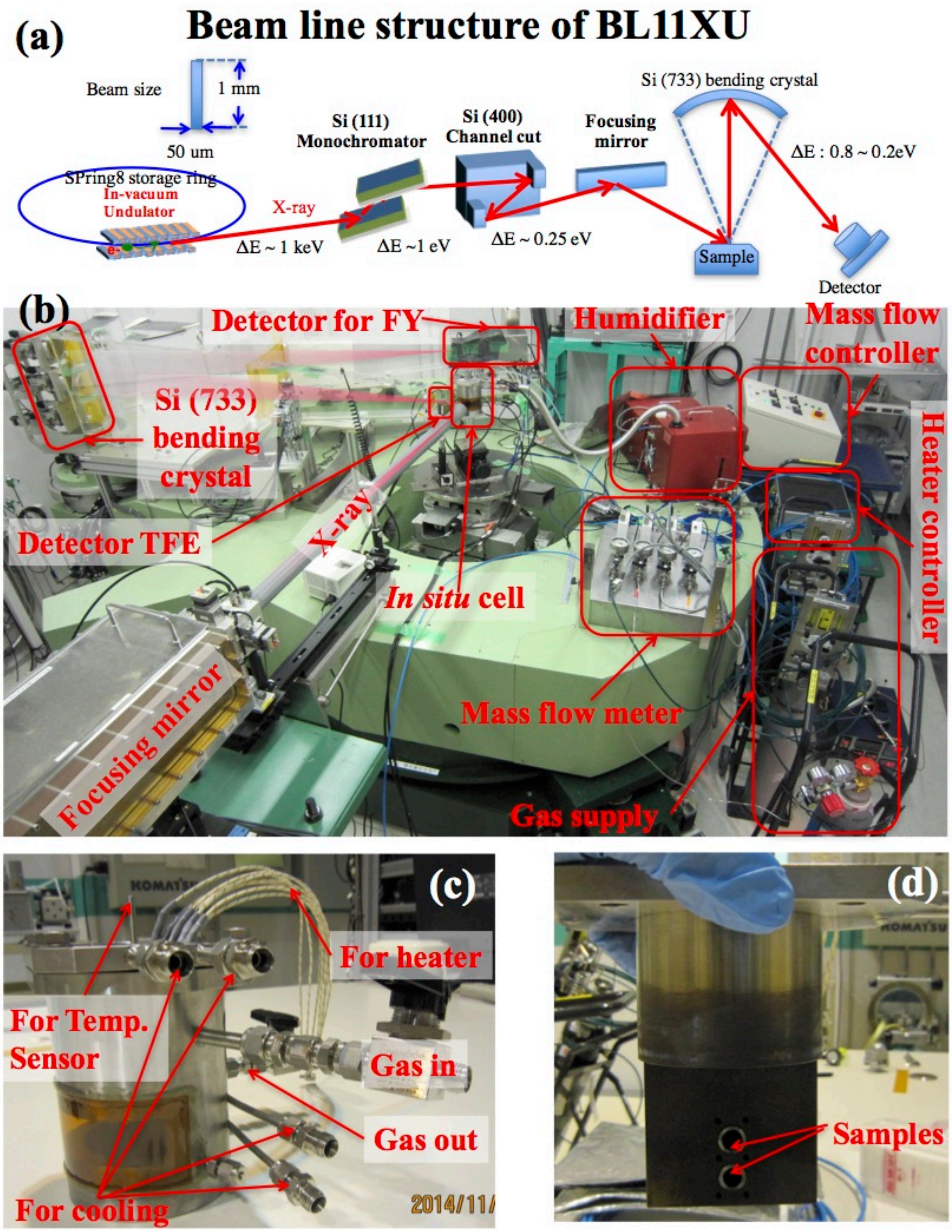

Fig. S6 Experimental setup for *in situ* HERFD-XAS experiment. (a) Optical layout of SPring-8 BL11XU. (b) Setup of the *in situ* cell during HERFD-XAS experiment. (c) *In situ* cell. (d) Sample holder.

References:

1. Langford, J. I. & Wilson, A. J. C. Scherrer after sixty years: A Survey and Some New Results in the Determination of Crystallite Size. *J. Appl. Crystallogr.* **11,** 102–113 (1978).

2. Safonova, O. V *et al.* Identification of CO Adsorption Sites in Supported Pt Catalysts Using High-energy-resolution Fluorescence Detection X-ray Spectroscopy. *J. Phys. Chem. B* **110,** 16162–16164 (2006).

3. Lytle, F. W., Wei, P. S. P., Greegor, R. B., Via, G. H. & Sinfelt, J. H. Effect of Chemical Environment on Magnitude of X-ray Absorption Resonance at L_{III} Edges. Studies on Metallic Elements, Compounds, and Catalysts. *J. Chem. Phys.* **70,** 4849–4855 (1979).

4. Horsley, J. A. Relationship Between the Area of $L_{2,3}$ X-Ray Absorption Edge Resonances and the d Orbital Occupancy in Compounds of Platinum and Iridium. *J. Chem. Phys.* **76,** 1451–1458 (1982).

5. Schulenburg, H., Durst, J., Müller, E., Wokaun, A. & Scherer, G. G. Real Surface Area Measurements of Pt_3Co/C Catalysts. *J. Electroanal. Chem.* **642,** 52–60 (2010).
